# High-Density 3D Printable Chipless RFID Tag with Structure of Passive Slot Rings

**DOI:** 10.3390/s19112535

**Published:** 2019-06-03

**Authors:** Zhonghua Ma, Yanfeng Jiang

**Affiliations:** 1 College of Information Engineering, Jimei University, Xiamen 361021, China; mzhxm@jmu.edu.cn; 2 College of IoT Engineering, Jiangnan University, Wuxi 214122, China

**Keywords:** chipless tag, radio frequency identification (RFID), radar cross-section (RCS), rectangular slot ring

## Abstract

A three-dimensional (3D) printable chipless radio frequency identification (RFID) tag, with high density and sensitivity, is proposed and fulfilled on insulator substrates. By printing a rectangular slot ring and designing specific geometry on the substrate, the printed structure shows high sensitivity in a resonant manner, with the benefits of high density and low cost. Considering the multiple rectangular rings with different sizes in a concentric distribution, a bit coding sequence can be observed in frequency spectra because of the corresponding different resonant frequencies aroused by the printed slots. In this way, the 3D printable chipless RFID tag can be fulfilled by adopting the structure of the rectangular slot ring on the insulated substrates. The main characteristics of the designed rectangular slot rings are verified on both flexible and solid substrates. A 12-bit chipless tag based on the slot ring structures is designed and implemented. The simulation and experiment results show good agreement on its characteristics. The frequency response reveals the fact that the 2th, 3th and 4th harmonic do not exist, which is a unique merit for improving the encoding capacity and the sensitivity of the corresponding reader. The electric field direction of the electromagnetic wave of the reader excitation tag is demonstrated to be wide, up to 90° on the tag horizontal plane, 30° on the vertical direction.

## 1. Introduction

Radio frequency identification (RFID) technology has been widely used in various fields, such as supply chains, transportation, personal identification, security systems, parking management and Smart-City application [1,2,3,4,5,6]. The RFID system is mainly composed of a reader, a tag and a data processing system [7], which can extract the stored information from the tag in a remote manner. The reader is mainly composed of a continuous wave generating circuit, a power amplifier circuit, a receiving circuit, a circulator, an antenna and a digital processing circuit. The tag consists of a tag chip and a tag antenna. The tag chip is composed of a voltage multiplier, a modulation/demodulator, a clock circuit, a register and a logic control circuit. The data processing system mainly refers to the background computer system. For the radio frequency tag, with much opportunity to take the place of current optical barcode, the most difficult issue on the replacement is how to cut down the tag’s cost to compete with the optical barcode while keeping its advantage of automatic remote reading [8]. Currently, main cost of the conventional RFID tag is related to the silicon chip in the tag [9]. So, one of the promising cost-down solutions is so-called chipless tag.

Until now, a lot of possible solutions on the chipless tag have been proposed in time-domain (TD) [10,11,12,13,14,15], space-domain [16,17,18] and frequency-domain (FD) [19,20,21,22,23,24].

The chipless tags operated in the time domain include SAW chipless tags and microstrip delay chipless tags [10,11,12,13,14,15]. The SAW chipless tag adopts a pair of passive dispersive SAW filters for pulse expansion and compression to improve anti-interference performance [10,11]. On this sense, its coding capacity is high. However, submicron etching technology is used on the high-cost piezoelectric substrate. So, its cost is high large area. The other chipless tag on time domain is the microstrip delay chipless tag, with the ID generated at higher frequency [12,13,14,15]. Its structure is a simple planar ones, which include antenna and ID generation circuit. The chipless tag based on transmission lines mainly used for detection, theft prevention and low-end commercial applications. However, its encoding capacity is limited to be only a few bits. 

Zhang et al. proposed the space angle-based chipless tag [16,17,18], in which the resonator was designed in V-shape and Y-shape. Its encoding was conducted by adjustment of the space angle between two arms of the V-shape and the Y-shape. The coding capacity can be above 3-bit. However, the structure is over-sensitive to the external factors and it is identified by measuring the scattered field in two orthogonal polarization directions. The method has high bit error rate (BER). 

The chipless tag based on frequency-domain is encoded by fulfillment of a resonator with specific geometry on the substrate. The spectral feedback of the UWB signal is changed accordingly. Its coding capacity depends on the number of the resonators and the available spectral scope. In References [19,20,24], a re-transmittable chipless tag is proposed for a microstrip transmission line loaded resonator. It requires two orthogonal UWB transceiving antennas connected by transmission lines. The microstrip resonators are loaded on both sides of the transmission line. The coding capacity of the chipless tag mainly depends on the number of the resonators and the available bandwidth. The literatures [21,22,23] showed a self-resonant chipless tag with reduced area because the two transmitting and receiving orthogonal ultra-wide band (UWB) antennas were neglected. Multi-slot structures were used in the chipless tag in the frequency-domain [25,26]. Islam proposed the circular ring slot and U-shape slot nested chipless tags [25,26]. Due to the symmetry of the slot ring structure, the tag can be rotated at any angle horizontally. So, the reader antenna can receive the coding information in a sensitive manner [25]. Islam also proposed a C-shaped slot nested chipless tag [26]. In order to reduce the interference, the adjacent C-shaped slots are placed on two nested patterns, respectively. The cost is high because of the large area of the tag. Meanwhile, the direction of the excitation electric field has to be consistent with the direction of the C-shaped opening, which limits its application.

Three-dimensional (3D) printing is a new emerging technique, which is suitable for the prototype fabrication in a rapid way. With the designed digital model files, a bondable material such as powdered metal or plastic is used to construct the designed object by layer-by-layer printing. It is also called additive manufacturing technique (AM), which can save material and lower the cost. 3D printing technology has been applied in medicine, sensors and electronics [27,28,29]. The technology is also used for printing inductors and capacitors in the circuit [30].

3D printing is a technology with a rapid production of 3D objects directly from digital CAD files. The 3D printing process is a bottom-up technology for 3D object fabrication. No milling or molding is needed anymore. In a 3D printing process, the material or silver paste is deposited in a successive way. Multiple layers of thin film are accumulated on the substrate to build up a solid 3D object. 3D printing process offers many advantages over traditional manufacturing techniques. It is a single step manufacture, with the merits of design freedom, low cost, easy customization and sustainability.

For the etching process, a mold is needed to confine the structure. We have used the method to fabricate a prototype of PCB tag [31]. The detail information can be found in the reference. The characteristics are similar to the 3D printing tag. However, its cost is relatively high. Its resolution is lower than that of the 3D printing tag. The substrate that can be used in the etching process is limited by many aspects, including its etching property, its thickness and its composites. It cannot be used on flexible substrate.

For the stencil/screen-printing, a mesh is needed for transferring the ink onto a substrate. The areas with the blocking stencil are impermeable to the ink. So, the blocking stencil is necessary.

Above all, the 3D printing process shows many merits compared with the traditional process. The designed structure is controlled by the digital file. The layers are defined by software that takes a series of digital cross-sections through CAD software. Descriptions of the slices are then sent to the 3D printer to construct the respective layers. The layers can be constructed in a number of ways depending on the 3D printer being used.

In this paper, a nested rectangular slot structure is designed and implemented by the fused deposition modeling (FDM) 3D printing technology. The tag can be printed on either insulating flexible substrates or solid substrates without any observable deterioration on the encoding rules and the spectral characteristics of the tag. The tag adopts a rectangular slot ring nesting structure with increased coding capacity without increasing the area of the tag. It has a compact structure and strong coding capacity.

## 2. The Principle of the Design

### 2.1. Working Principle of Chipless Tag RFID System

**Figure 1:**
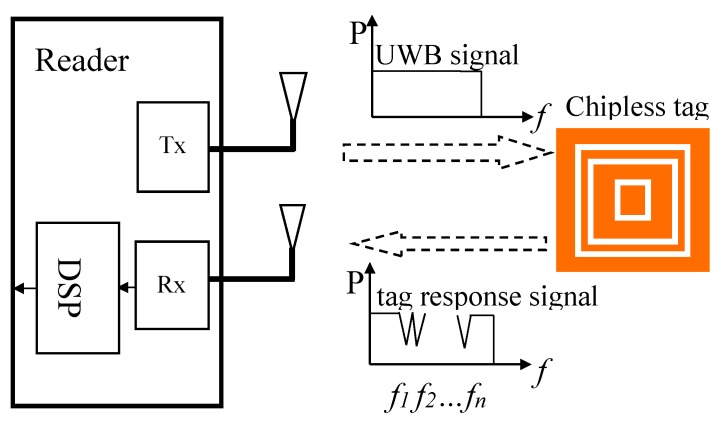
Working principle of RFID system, in which UWB signal is transmitted by the reader. The tag receives the signal and submits its reaction in resonant way. The reader receives the encoded signal.

The working principle of the RFID system with chipless tag as shown in Figure 1. The UWB signal is transmitted to the tag through the radiation of the reader transmitting antenna. The feedback UWB spectrum from the reader is tuned accordingly because of the resonant structure existing on the tag. In this way, the data carried on the tag is encoded into the UWB signal and backscattered to the reader receiving antenna.

**Figure 2:**
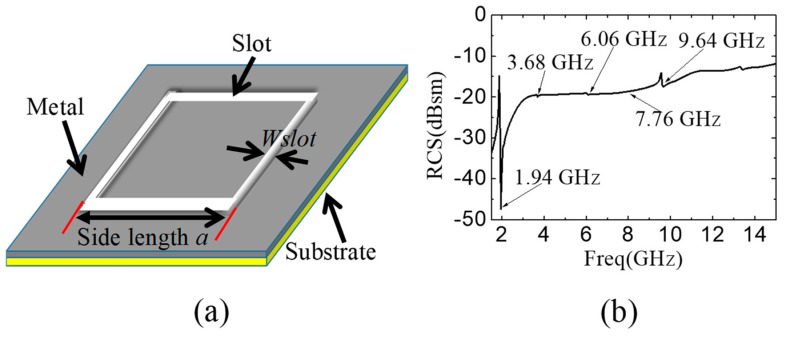
(**a**) Schematic of slot ring embedded on insulated substrate. The substrate is insulator, without any conductor on back side. (**b**) Spectrum of RCS of the single slot. The side length of rectangle a = 30 mm. The width of slot *W_slot_* = 0.5 mm. Its resonant frequency is 1.94 GHz.

The structure in this paper is the rectangular slot ring printed on insulated substrate, which is depicted in Figure 2a. It can be seen that the resonator structure is the concave-down slot ones, which is obviously different from the strip structure [32]. In our design, the coverage of the deposited structure on the substrate is above 95%. On the contrary, the metal coverage of the strip structure [32] is below 5%. The designed tag is implemented by 3-D printing technology. The nanoparticle silver paste is used in the 3D printer (Voxel8) as conductive material. Nanoparticle silver paste is provided by Silver Paste-SP3012 of Guangdong Nanhai ETEB Technology Co., LTD. The printing system is Voxel8, manufactured by Somerville, USA. After the subsequent heat treatment process, the curing temperature is set to 200 ℃ and the bulk resistivity of the nanoparticle silver paste reaches 3 μΩ·cm. The adopted substrate here is Teflon woven glass fabric copper-clad laminates (F4BM), with thickness 1 mm, permittivity 2.2 and loss tangent 0.0007. 

### 2.2. 3D Printing Technology

The designed 3D model file is saved in the STeroLithography (STL) software and converted into a 3D printing file. The printing path is set up as the following: on the main menu of the software, the type of the printer is selected. Here the 3D printer is a triple-head Voxel8 purchased from Voxel8, USA. Also, the printing material is chosen based on the design. The STL file is opened for the slicing configuration. The thickness of the slice is set to be 0.05 mm. The sliced file is saved in gcode format. The structure and the printing material information of the model are converted into a series of 2D image files. 

The printer’s curing temperature was set to be 200 ℃ for curing the conductive nano-silver paste extruded from the nozzle. The best conductivity can be obtained at this temperature. Moreover, it is within the toleration temperature of the F4BM dielectric substrate. The F4BM dielectric substrate was loaded onto the 3D printing platform. After loading, the leveling was adjusted. The conductive nano silver paste was filled in the 3D printer. The molding direction and the pattern were determined by the designed modeling. Certain distance was kept between the nozzle and the printing platform to prevent the nozzle from blocking. The printing speed was estimated to be 8 mm^3^/s.

Here we present a new paradigm in 3D printing technology with the formation of the new simple, low-cost conductive silver material from easily available starting materials. The material is used in conjunction with Voxel8 3D printer to produce the RFID tags. Standard printing settings were used and no modifications to the printer were required.

In order to formulate a conductive material to be used by Voxel8 3D printer, the conductive silver filler is chosen. The melt viscosity of the material is also considered. The filler ratio is adjusted not only to satisfy the high volume but also to enable the material to flow out of the heated extrusion nozzle of the 3D printer easily. Multiple rounds of trial-and-errors were conducted to optimize the experiment data.

### 2.3. Characteristic of the Rectangular Slot Ring Resonator

The relationship between the resonant frequency and the side length of the rectangular slot ring is defined as [33]:

(1) $$f=\frac{C}{4a}\sqrt{\frac{2}{\varepsilon_{r}+1}}$$

where *C* is the speed of light in free space, $\varepsilon_{r}$ is the relative dielectric constant of the substrate and *a* is the side length of the rectangular slot ring. The equation does not take into account the effects of slot width and dielectric thickness on the resonant frequency.

First of all, we need to verify the resonant characteristic of the designed slot. For a slot with side length 30 mm, the resonance frequency is simulated to be 1.94 GHz, as shown in Figure 2b, which is simulated by High-Frequency Structure Simulator (HFSS^TM^). The nanoparticle silver paste with the resistivity 3 μΩ·cm [34] and the thickness 100 μm is adopted to print the designed RFID tag on F4BM substrate. It is observed that the 2th, 3th and 4th harmonics do not appear in the output spectrum but the 5th does, as shown in Figure 2b. Only the fundamental resonant and the fifth harmonic can be generated and observed. Due to the symmetrical and continuous structure of the rectangular slot ring resonator, the current flow paths for the second, the third and the fourth harmonics cannot be generated. This is the main reason that they cannot be observed in the RCS spectra. Only the fundamental frequency resonant path and the fifth harmonic path can be generated and observed. Therefore, the unique property shows one of the design rules on the upper limit of the resonant frequency when multiple slots are considered. Based on Equation (1), the calculated resonant frequency is 1.975 GHz when the side length is 30 mm. A slight difference between the theoretical calculation and simulation do exist but will not influence the data coding. The slight discrepancy mainly comes from the ignorance of the width of slot and the thickness of the dielectric substrate during calculation.

When a notch is shown in the RCS spectrum of the corresponding rectangular slot, the logic state of the code is “1”; while there is not a notch, the logic state of the code is “0.” The different sizes of rectangular slot rings correspond to the different resonant frequency. So, a series of notches are shown at different positions of the spectra. By adding or removing the slot ring resonators of different sizes, the notches of the spectrum are shown in a coding manner. 

**Figure 3:**
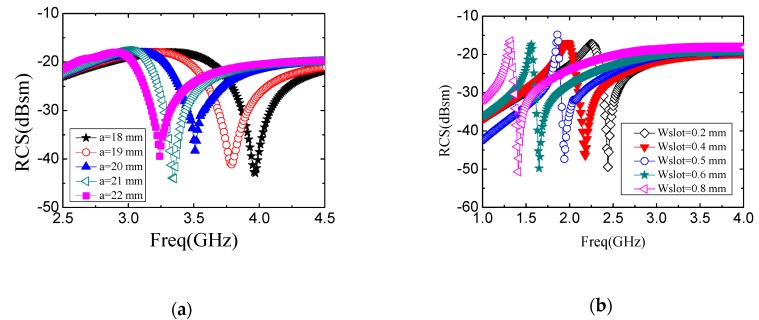
(**a**) Relationship of side length and resonant frequency with *W_slot_* = 0.5 mm. (**b**) Relationship between *W_slot_* and resonant frequency with a = 30 mm.

Figure 3a shows the simulation results of the relationship between the resonant frequency and the side length. The resonant frequency is changed from 3.97 GHz to 3.24 GHz with the side length increasing from 18 mm to 22 mm.

The influence of the slot width on the resonant frequency is also investigated, as shown in Figure 3b, in which the side length is fixed to be 30 mm. The slot width is changed from 0.2 mm to 0.8 mm. When the slot width is 0.5 mm, the resonant frequency is 1.94 GHz. When the slot width is 0.6 mm, the resonant frequency is changed to be 1.66 GHz. While the slot width is 0.4 mm, the resonant frequency is increased to be 2.19 GHz accordingly. It can be concluded that the resonant frequency drops with the increment of the slot width. Although increasing the slot width can shrink down the operation band, a suitable width should be determined based on the requirement on the tag area and the consequent cost.

### 2.4. Chipless Tag of the Rectangular Slot Rings

**Figure 4:**
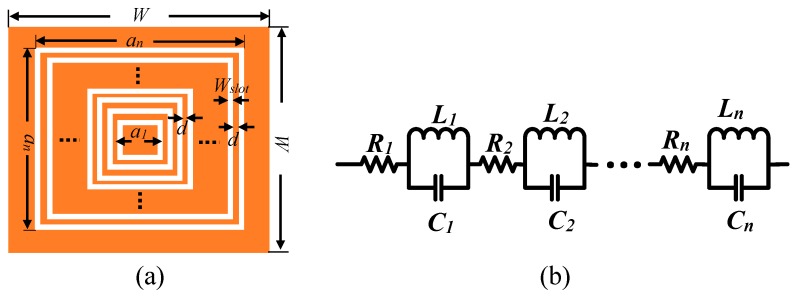
(**a**) Diagram of the designed concentric rectangular slots printed on substrate; (**b**) Equivalent circuits of rectangular slot ring resonators. Each rectangular slot ring is depicted as a LC shunt circuit.

The above analysis is suitable for the printed single slot. To create a multi-resonant structure for the purpose of multi-bits coding, the multiple slots need to be printed in a compact manner. Here a concentric contour is designed to include multiple rectangular slots with different sizes while keeping the highest compact density, as shown in Figure 4a. The key parameters include the width of slot *W_slot_* and the gap distance of adjacent slots d. For the structure with n-bit resonant slots, the size of the tag can be expressed as:

(2) $$a_{n}=a_{1}+2(d+W_{slot})\times n$$

where *a*_1_ is the size of primary resonant slot.

The macro-model of the tag is shown in Figure 4b. It shows the equivalent circuit of the proposed resonant slot-patch RFID, in which LC coupled resonator is used to establish the resonant frequency. R indicates the attenuation of the UWB signal. C represents the capacitance of the sealed slot. Its value is proportional to the total length of the slot while being inversely proportional to the width of the slot. L is used to describe the inductance of the metallic strips and its value is also proportional to the total length of the metallic strips while being inversely proportional to the width of the strips. In our design, the equivalent capacitance and inductance are increased significantly because of the convoluted structure that makes both the slot dipole and the metallic strips longer.

**Figure 5:**
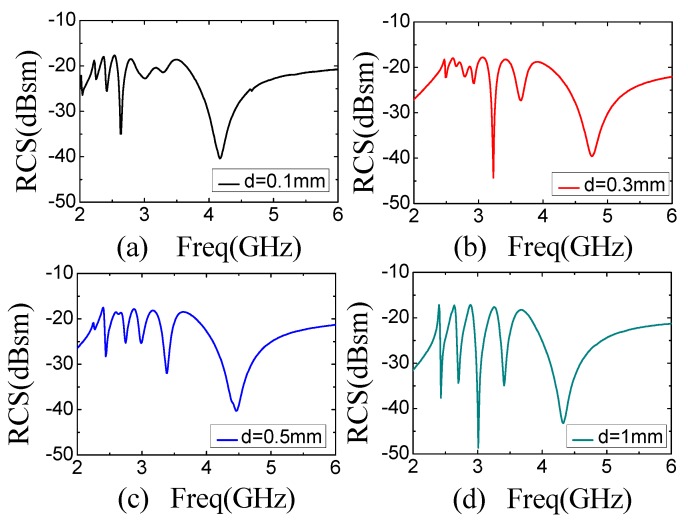
RCS results of five Rectangular Slot Rings with different *d* while the slot width is fixed to be 0.5 mm. (**a**) d = 0.1 mm (**b**) d = 0.3 mm (**c**) d = 0.5 mm (**d**) d = 0.9 mm.

For the designed concentric structure in Figure 4a, one important parameter is the gap between two adjacent slots, d. The gap here corresponds to the convex part on the substrate. To investigate the influence of the gap on the resonant property, a simulation is conducted on the designed tags with gap values varied from 0.1 mm to 0.9 mm while the slot width fixed to be 0.5 mm. The results are shown in Figure 5. It shows that the coding results are sensitive to the gaps. For the designed structure, the coding of resonant frequencies can be distinguished clearly when the gap is larger than 0.5 mm, as shown in Figure 5c,d, while obvious data missing occurs when the gap is smaller than 0.5 mm, as shown in Figure 5a,b. The phenomenon can be explained by the coupling effect, in which the effect of the adjacent slot rings is more obvious at smaller gap because the interference appears stronger.

So, based on above analysis, the thumb rules for the 3D printed chipless tag based on the designed concentric slots on insulator substrate include:

(1) The upper resonant frequency is limited by the 5th harmonic frequency of the fundamental frequency.

(2) The slot width should be larger than 0.3 mm.

(3) The gap among slots should be larger than 0.5 mm to guarantee the coding quality.

## 3. Design and Fabrication of 12-bit Chipless Tag Printed on F4BM Substrate

To fulfill a 12-bit-tag with the designed structure on F4BM substrate, key geometry parameters are shown in Table 1.

**Figure 6:**
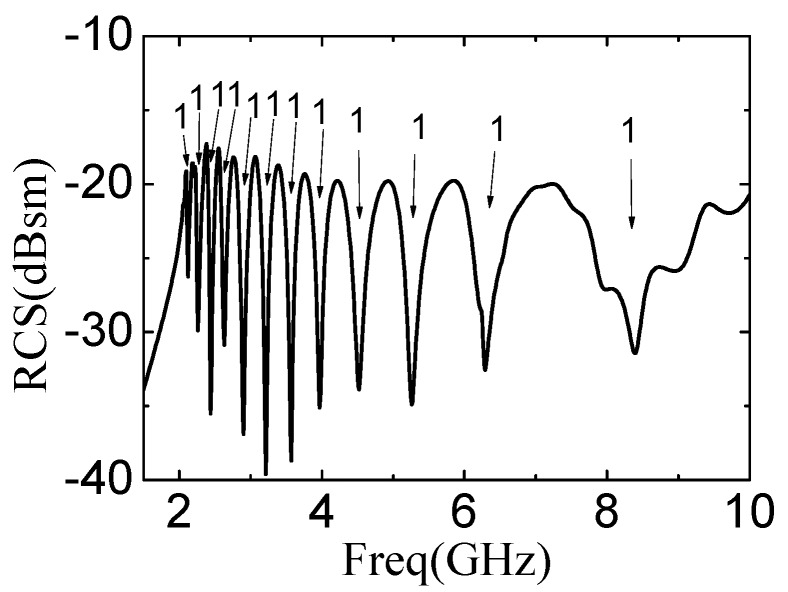
Frequency spectra of the 12-bit-tag on F4BM substrate. The thickness of substrate is 1 mm.

Figure 6 shows the simulation result of the designed 12-bit-tag with coding (111111111111) when thickness of printed conductor is 100 μm and the thickness of substrate is 1 mm. The tag of ID (111111111111) is used as a reference tag in this paper. Other tags with different codes are formed based on the reference tag by adding and removing the corresponding bit slot rings. It can be seen in Figure 6 that the resonant frequencies of the reference tag are distinguished clearly, with frequency ranging from 2.12 GHz to 8.39 GHz, showing promising property as candidate of the chipless tag.

**Figure 7:**
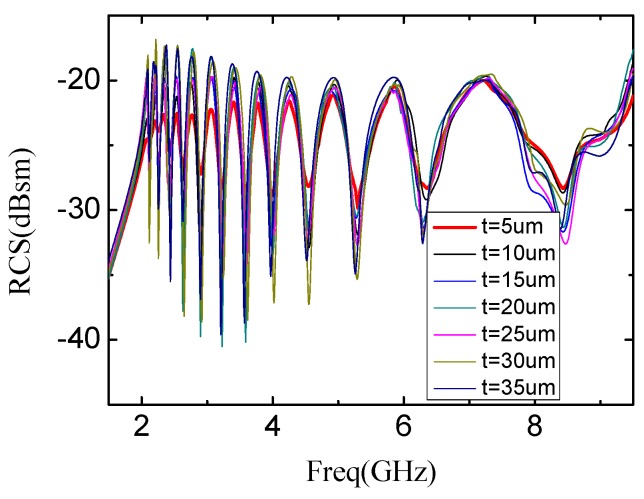
RCS results of Rectangular Slot Rings with different thicknesses of silver nanoparticle conductive ink layer.

Figure 7 shows the simulated resonant spectra of the reference tag, with the thickness of the silver nanoparticle conductive paste layer varied from 5 μm to 35 μm with the increment step 5 μm. It can be seen that the resonant frequencies of every slots are almost fixed although the thickness of the conductivity nanoparticle silver paste is changed from 5 μm to 35 μm. This means the resonant frequency of the printed tag is robust to the printing thickness. The twelve notches of the spectrum signatures clearly demonstrate the correctness of the encoding state. However, the notch of spectra is turned to be shallower when the thickness of the silver nanoparticle conductive paste layer is decreased. When the thickness of conductive nanoparticle silver paste layer is 5 μm, the notch is turned to be too shallow to be correctly decoded.

**Figure 8:**
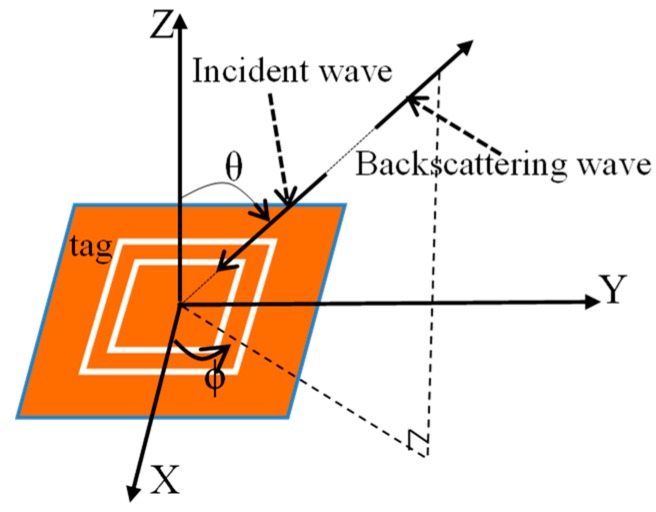
Schematic of incident angle of the signal θ relative to the vertical orientation, angle of horizontal rotation φ defined on the tag.

**Figure 9:**
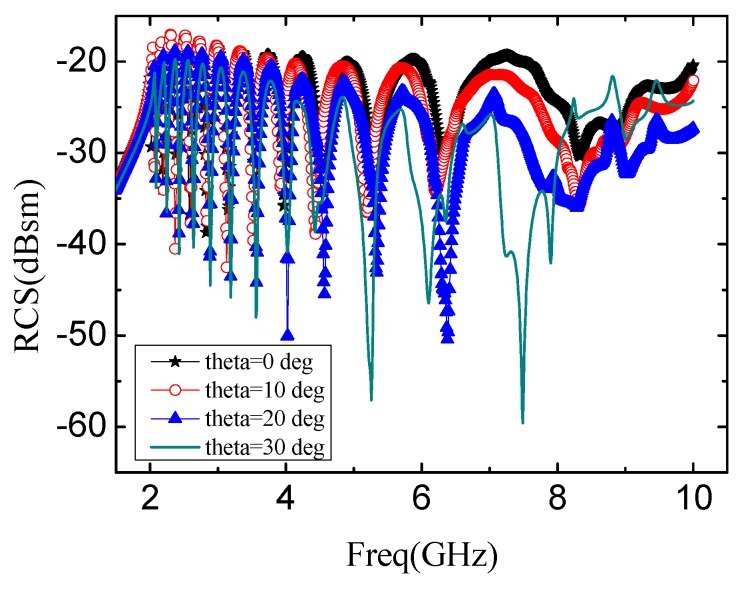
The spectrum of altering θ from 0° to 60° when φ = 0°. The substrate is F4BM.

**Figure 10:**
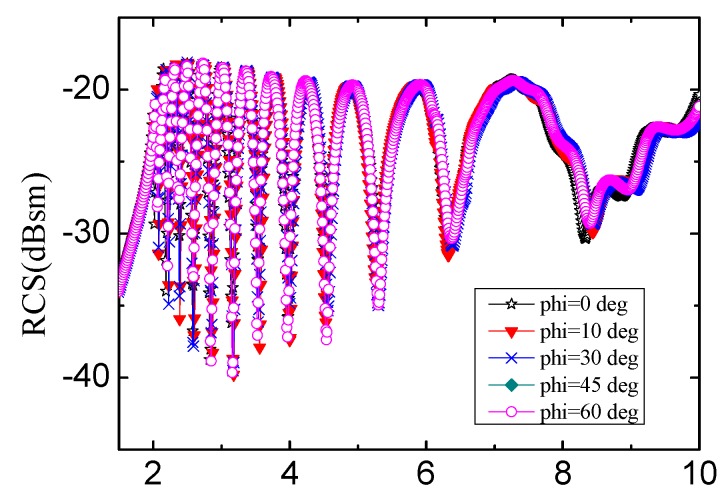
The spectra corresponding to φ from 0° to 60° when θ = 20°. The substrate is F4BM.

The diagram in Figure 8 defines the angles of incident wave and reflection wave, in which θ denotes the incident angle of the signal relative to the vertical orientation and φ is the angle of horizontal rotation. The spectra obtained by changing θ and φ of incident wave are shown in Figure 9 and Figure 10, separately.

Figure 9 shows the RCS results while θ changing from 0° to 30° and φ = 0°. The transmitted code keeps correctly when θ < 30°. However, when θ = 30°, error data appears because of the occurrence of unusual offset. This reveals that the tag cannot rotate exceeding 30° on the vertical direction.

Figure 10 shows the RCS curves on condition that θ = 20° and φ changing from 0° to 60°. The reader can receive data from tag correctly when the tag rotating on the horizontal direction from 0° to 360°, demonstrating directly that the tag shows promising properties acting as RFID since the data in tag can be easily read in wide angle.

The above analysis does not include the influence of the thickness of the substrate. To correctly understand its influence, the simulation is conducted with different thicknesses of the substrate, including 0.5, 1 and 1.5 mm, respectively. The corresponding resonant frequencies are listed in Table 2. It can be seen that there is slight difference on the slot ring with same size but with different substrate thickness. But the coding qualities are all fine on the three substrates.

**Figure 11:**
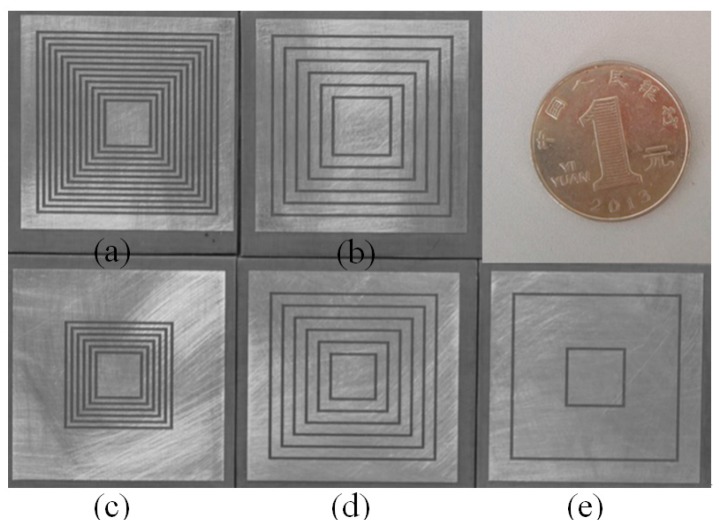
Photograph of the Rectangular slot ring tags with different IDs. (**a**) ‘111111111111′, (**b**) ‘101010101010′, (**c**) ‘000000111111′, (**d**) ‘010101010101′, (**e**) ‘010000000010′.

A variety of tags, with codes (111111111111), (101010101010), (010101010101), (000000111111) and (01000000010), are 3D printed by adopting the above design rules. Figure 11 shows the photos of the 3D printed tags. To get a clear picture on the actual size, a coin is also included in Figure 11.

For example: Figure 11a is a reference tag. In Figure 11b, some slot rings are removed. The RCS spectra characteristic of those removing resonators are disappeared. The RCS spectra characteristic of the existing resonators still exist and its logic state is “1.” This combination of presence and absence of rectangular slot ring resonators constitutes the encoding of the chipless tag.

**Figure 12:**
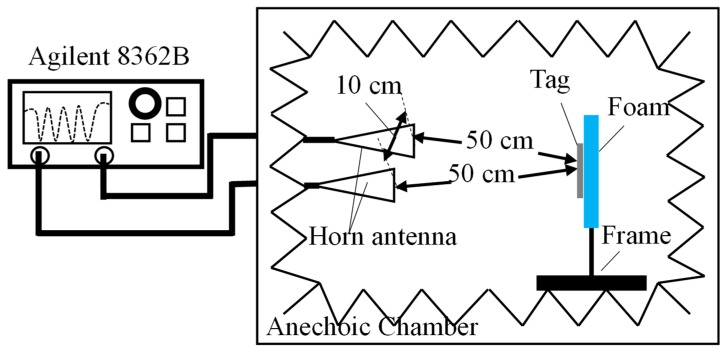
Block diagram of the measurement setup. Distance between two horn antennas is 10 cm and distance between horn antenna and tag is 50 cm.

To verify the above analysis experimentally, the actual measurement is conducted in the anechoic chamber. Figure 12 shows the facilities to characterize the fabricated tags, including vector network analyzer (VNA) (model: Agilent E8362B) and two horn antennas (model: LB-8180-NF and LB-10180-NF) acting as transmitting and receiving antennas. The VNA can replace the chipless RFID reader in the laboratory. The distance between Tx and Rx horn antennas connecting the VNA is 10 cm. The chipless tag is fixed on the foam of the frame. The distance between the two horn antennas and the chipless tag is 50 cm.

The Ref [35] indicated that an accurate RCS value of the tag can be obtained using Equation (3):

(3) $$\sigma^{tag}={\left[\frac{s_{21}^{tag}-s_{21}^{iso}}{s_{21}^{ref}-s_{21}^{iso}}\right]}^{2}\cdot \sigma^{ref}$$

where $\sigma^{ref}$ is the complex RCS value of the metallic reference plate obtained with analytical formula or simulation. Firstly, the background items that produce static noise is removed. $S_{21}^{iso}$ is the isolation of two-horn antennas measured without any tag. $S_{21}^{ref}$ is measured when the identification item is present. Finally, $S_{21}^{tag}$ is measured when the tag is adhered on the identification item.

**Figure 13:**
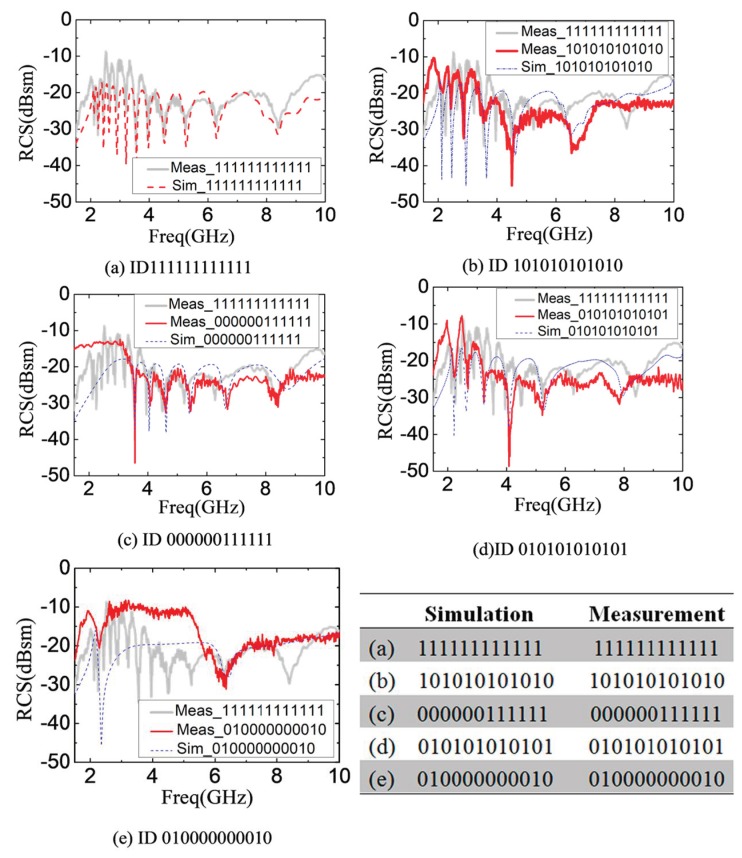
RCS magnitude for twelve Rectangular Slot Rings with (**a**) ID 111111111111 (**b**) ID 101010101010 (**c**) ID 000000111111 (**d**) ID 010101010101 (**e**) ID 0100000010.

Figure 13a–e show the characterization results corresponding to the tags with ID (1111111111), (101010101010), (000000111111), (010101010101) and (010000000010), including both simulation results and experimental results. For the tag coded 111111111111, its result is shown in Figure 13a. The resonant frequency points include 2.12, 2.26, 2.44, 2.63, 2.9, 3.21, 3.57, 3.97, 4.52, 5.26, 6.29 and 8.39 GHz. Figure 13a shows the measurement and simulation results of the tag ID111111111111. When the 12 resonators are present, the coding states are all “1.” That is, there are 12 notches in the spectral structure. The tag ID111111111111 is used as the reference tag for the evaluation of the corresponding states of the other tags. If the measured tag has the notch on the same position of the reference tag, it is labeled as the logic state “1.” On the contrary, if the measured tag has not the notch on the same position of the reference tag, it is the logic state “0.” 

Figure 13b–e indicate the result of simulation and measurement for the other coding tags. The frequency points of the logic ‘1′ and logic ‘0′ are in accordance to the reference tag ID(111111111111) of Figure 6. It is worth noting that results of simulation and measurement have obvious deviation at the high-frequency-range because of the geometry error on the printing of the smaller slot rings. However, the feasibility of our design can be verified. In practical application, UWB disc monopole antenna [20,36] and CPW-fed quasi-circular monopole antenna [37] are used as the reader antennas.

**Figure 14:**
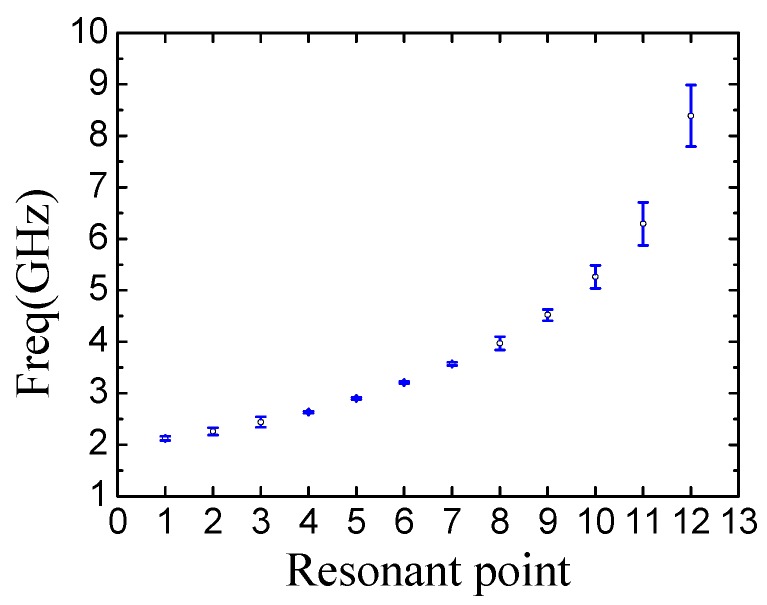
Statistic results on the distribution of the errors of the twelve resonant points.

Figure 14 shows the statistic results of the measured errors corresponding to the twelve rectangular slot rings. The minimum error is 0.02 GHz and the maximum is 0.6 GHz. It can be seen that the measurement errors are increased gradually with the resonant frequency increasing. On the one hand, the minimum side length of the rectangular slot resonant ring corresponds with the maximum error. The deviation of the resonant frequency is increased rapidly although there is only slight change in the dimension. On the other hand, according to Equation (1), compared with longer side, the shorter side of the rectangular slot ring corresponds to larger variation of resonance frequency. On the sense, precise fabrication is necessary in future industrial fabrication if high accuracy of resonant frequency is required in RFID identification system.

## 4. Discussion

**Figure 15:**
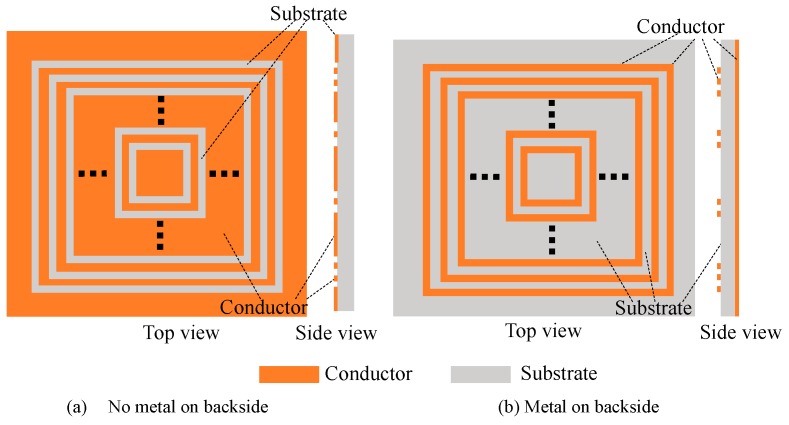
(**a**) Schematic of the designed tag, with top view and cross section, showing slot ring structure; (**b**) The structure proposed by Costa [38,39], using microstrip as resonator, with ground conductor on backside.

One of the merits of our design is the coverage of the 3D printing conductive material on the surface. To get a clear comparison with other similar work, the schematic pictures of our design and the strip structure [38,39] are shown in Figure 15. Our work is illustrated in Figure 15a. Filippo Costa et al. [38,39] used rectangular structure as resonator to design chipless tag, which structure is shown in Figure 15b. In our design, the coverage of the deposited conductive structure on the substrate is above 95%. On the contrary, the coverage of the strip structure [38,39] is below 5%. It can be seen that Costa’s structure used rectangular microstrip as resonant part. Most of conductor on surface was etched away. Key parameters comparison of Costa’s and this design are shown in Table 3.

For our design, as shown in Figure 15a, the tag is printed on the surface area. The resonant property can be observed clearly although no back-metal included. This is very unique when it is compared with other groups’ results. For other results, back-metal is necessary to keep resonant property. On this sense, we believe the designed tag in this paper shows good resonant property including high sensitivity, narrow bandwidth, no interference, RCS amplitude consistency and fixed resonance frequency point. In our design, the depth of the spectrum notch is responsible for the high sensitivity. The resonant bandwidth determines its spectrum utilization. The steepness and smoothness of notch determines the small interference. The fixed resonant frequency point determines the correctness of the coding and decoding.

The good resonant property can be observed on the printed tag without the back metal. The tag in this paper can be 3D printed directly on the surface of the items without any sacrifice on its properties. For Costa’s structure [38,39], it cannot be 3D printed because the back metal is necessary. By comparing the two structures in Figure 15, our design is easier to be fabricated, while keeping bigger scatter cross area.

Table 4 shows the comparisons among different chipless tags, including the properties of excitation, harmonic, ground, coding density, capacity bits and range. It can be seen that the 3D-printed work shows the most promising properties among all the listed results. The C-shaped slot and the C-shaped microstrip structures are limited by the direction of the excitation. On this sense, the direction of the excitation must be aligned with the opening orientation of the C-shaped slot resonator. On the other hand, the excitation must be orthogonal with the opening direction of the C-shaped microstrip resonator. Although the nested C-shaped slot and the nested C-shaped microstrip chipless tags have a coding density of 7 bit/cm^2^ and 3.3 bits/cm^2^, they are limited by the third harmonic. So, the actual number of code bits is limited by the third harmonic frequency. The other obvious shortcoming is the error of the circular slot and the circular microstrip ring. To overcome the error, the precision of the designed geometries is highly required. Moreover, a ground plate is mandatory required on the back side for the rectangular microstrip ring structure of the chipless tag substrate. 

Although the circular microstrip ring and the circular slot ring have not the harmonic interference, this structure is not easy to resonate and the regular spectral characteristics appear for a specific structure. It is not suitable for general application. The rectangular slot ring that we proposed shows significant spectral characteristics on different media under different dielectric thicknesses. In summary, the chipless tag of the rectangular slot ring resonator proposed in this paper has the following advantages. This chipless tag is printed by the advanced 3D printer with the high processing precision. The encoding is not limited by the second harmonic, the third harmonic and the forth harmonic. So, the coding frequency band is wide. The electric field can be incident from any direction, without limitation on the orientation. The ground plate is not required on the back side. So, the full-printing can be realized on any substrates. It has large coding capacity and the long reading distance.

**Figure 16:**
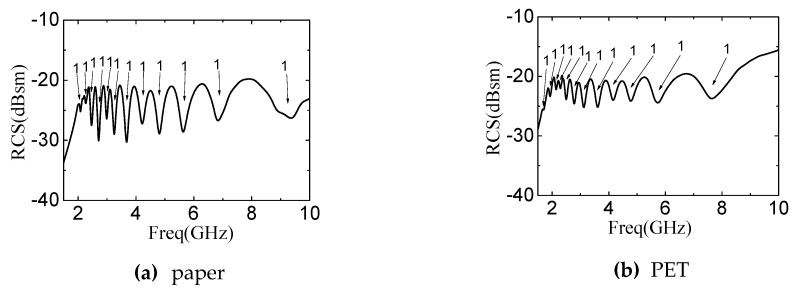
The simulation spectra of the twelve rectangular slot rings when the substrates are paper and PET, respectively.

We also demonstrate the feasibility of our design on other flexible substrates, such as paper and polyethylene terephthalate (PET). The key parameters of substrates are shown in Table 5. The simulation results are show in Figure 16. The parameters of paper include the thickness 1 mm, the relative dielectric constant 2.25 and the loss tangent 0.045. The parameters of PET used in simulation include the following: The thickness 1 mm, the relative dielectric constant 3.4 and the loss tangent 0.07. The spectra signature of the paper substrates can show the coding capability of the tag clearly. The corresponding resonant point of the largest rectangular slot ring in Figure 16b is unclearly and the other resonant points are still show correctly coding state. It can produce the coding error, therefore, the structure size of chipless tag must be adjusted.

**Figure 17:**
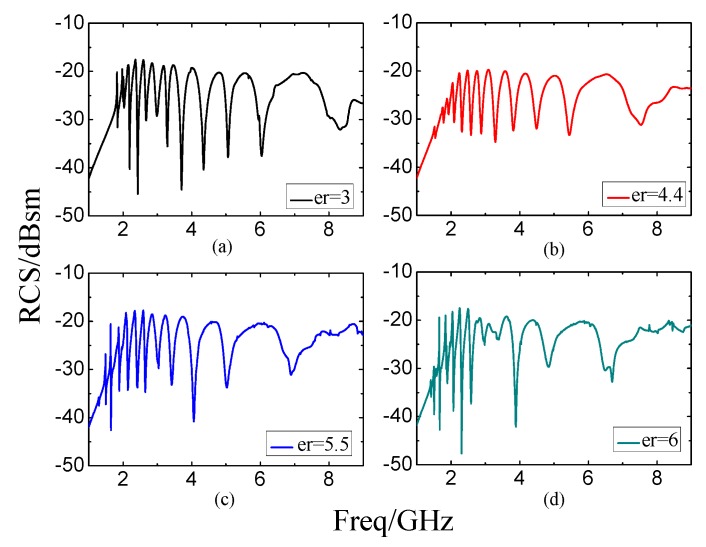
The simulation spectra of the twelve Rectangular Slot Rings when the FR4 substrate has different relative dielectric constants (*ε_r_*), respectively. (**a**) *ε_r_* = 3, (**b**) *ε_r_* = 4.4, (**c**) *ε_r_* = 5.5, (**d**) *ε_r_* = 6.

Figure 17 shows the simulation results of the designed 12-bit reference tag with coding (111111111111) on substrates with different relative dielectrics. Figure 17a,c,d show that the encode capacities are 1.84 bits/GHz, 2.14 bits/GHz and 2.35 bits/GHz when the relative dielectrics are 3, 5.5 and 6, separately. This means that the narrower operation band corresponds to the substrate with larger permittivity, which is accompanied by larger encode capacity. Figure 17b shows the result on FR4 substrates. The resonance point of the highest frequency is 7.54 GHz while the lowest frequency is 1.54 GHz. The encoded capacity is 2 bits/GHz.

The twelve resonant frequencies are distinguished from each other in a clear manner. As far as authors know, this is the first time to fulfill a 3D printed tag without any requirement on the substrate, showing a wide application potential for our proposed RFID tag. This is a practical RFID tag with the potential ability to take the place of the current optical barcode.

Islam et al. also proposed the C-shaped slot chipless tag [26], in which the direction of excitation electric field should be aligned with the opening of C-shape. So, the application of the tag is limited because of the requirement for the alignment. The two corners of the resonator should be short-connected with suitable conductor when the logic is changed from “1” to “0.” On the scenario that the C-shape slot is adopted, any short-connection of the corners will have obvious influence on the resonant frequencies of the three straight line slots. The coding will be complex and not suitable for application.

The encoding capacity is increased by adding the concentric rectangular slot rings. Simultaneously, the width of the slot is reduced to shrink down the area of tag. Preradovic et al. proposed the spiral resonator chipless tag [43] but the structure is complex. The tag needs two orthogonal Tx-Rx antennas and the corresponding Tx-Rx antennas must be aligned with each other. Otherwise, the reader will not receive the encoding signal. Vena et al. proposed C-shaped microstrip resonator chipless tag [22]. There is a critical constraint that the electric field excitation must perpendicular to the opening direction.

In our design, the 2th, 3th and 4th harmonics don‘t influence the coding condition. The encode capacity is 1.9 bits/GHz. The excitation can be introduced from any orientation. The most interesting point of our design is that no ground plane (back metal) is required anymore. In this way, our design can be printed on any substrate and fulfill the same function as that of the barcode.

The conventional RFID reader operates in narrowband. It uses ASK demodulation algorithm in time domain. So, the cost is lower. The chipless RFID reader works in the UWB. The encoded data is extracted from the spectra of the backscatter signal. On this sense, the reader part is more complex that of the conventional RFID. On this point, the cost of the chipless RFID is higher. However, the cost of the tag used in the chipless RFID is much lower. It is also much easier to be used.

**Figure 18:**
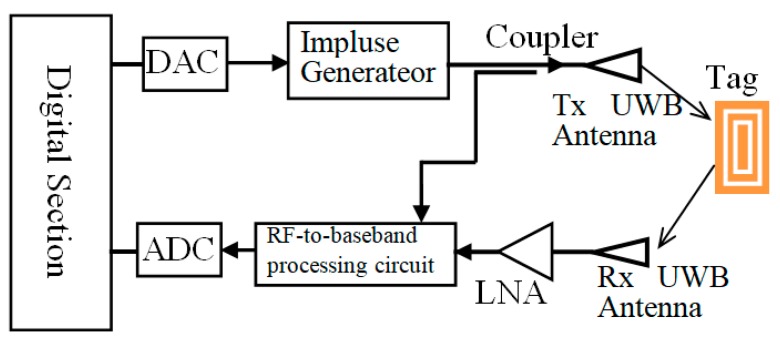
Block diagram of the chipless reader.

Figure 18 is the block diagram of the chipless RFID reader. The UWB interrogation is generated by the impulse generator, which is controlled by the digital circuit. D/A convertor is used to transfer signal into digital ones. The interrogation signal is transmitted to the chipless tag through the UWB transmitting antenna. The backscattered signal is received by the UWB receiving antenna of the reader. The RF-to baseband processing circuit demodulates the encoding UWB signal from the LNA. After ADC conversion, the information in the tag is obtained by the signal processing technique. 

Although 3D printer and chipless UWB reader are somehow expensive, the chipless tag can be used for large scale fabrication. The cost of a chipless tag for 3D printing can be reduced to less than 0.1 cents. For the comparison, the cost of a chipless RFID system depends primarily on the large number of tags. The cost of a barcode is about 0.05 cents, while the cost of a conventional RFID tag is 3 cents [44]. Although the 3D printed RFID is slightly more expensive than that of a barcode, the multifunctions of the 3D printed RFID, such as automatic recognition, contactless reading, automatic tracking and working in extreme environments and so forth, make it a promising candidate for application.

For the RFID system, the security and encryption is highly required to protect the information [45,46,47,48]. For the RFID with active chip inside, the encryption can be performed in the chip. For the chipless tag, the information cannot be secured directly on the tag because of lacking the necessary hardware in the tag. A future study should be investigated to find an approach to protect the security of the chipless tag.

## 5. Conclusions

A novel compact chipless RFID, utilizing the concave-down slot on F4BM substrate, is proposed and demonstrated in this paper by 3D printing technology, with the benefit of high density and high sensitivity. This is the first time to show a real 3D printable RFID tag. 

The tag with 12-bit coding capacity is realized in the 2 GHz to 9 GHz bandwidth. The correct data signal can be received by reader at 50 cm from the tag. Simulation and measurement results show good agreement. The tag is low cost and convenient for coding. It can be printed easily on flexible and solid substrates, with promising future to take the place of the incumbent optical barcode.

## Figures and Tables

**Table 1:** Key geometry parameters of 12-bit-tag printed on F4BM substrate.

Parameter	Value
Side length of primary slot (*a*_1_)	8 mm
Width of slot (*W_slot_*)	0.5 mm
Distance of the adjacent slots (*d*)	0.5 mm
The outmost side length of outmost slot (*a*_12_)	30 mm
Thickness of silver nanoparticle ink	35 μm
Area of tag	35 × 35 mm

**Table 2:** Resonance frequency of different substrate thickness.

Thickness	1	2	3	4	5	6	7	8	9	10	11	12
h = 0.5 mm	2.14	2.31	2.6	2.71	2.97	3.29	3.68	4.13	4.75	5.58	6.72	8.92
h = 1 mm	2.12	2.26	2.44	2.63	2.9	3.21	3.57	3.97	4.52	5.26	6.29	8.39
h = 1.5 mm	2.07	2.19	2.35	2.56	2.83	3.15	3.53	3.93	4.48	5.21	6.25	8.29

**Table 3:** The Comparison between Costa’s and this work.

Kind	Structure	Ground	Notch depth	Manufacture	RCS Amplitude Consistency
Costa’s	Rectangular strip ring	yes	−18 dB	etch	poor
This work	Rectangular slot ring	no	−20 dB	3D printing	good

**Table 4:** Comparisons of different structures’ characteristic.

Structure	Excitation	Harmonic	Ground	Coding Density (bit/cm^2^)	Capacity (bits/GHz)	Range (cm)
C-shaped slot [26,40]	limit	3th	no	>7	1.45	50
C-shaped microstrip [41]	limit	3th	no	3.3	0.46	65
Circular slot [25]	no	no	no	4.18	2	20
Circular microstrip ring [42]	no	no	no	2.1	2	40
Rectangular microstrip ring [38,39]	no	5th	yes	0.49	1.25	50–55
3D-printed rectangular slot ring (this work)	no	5th	no	0.98	1.9	50

**Table 5:** Parameters of dielectric materials.

Substrate	Relative Dielectric Constant	Loss Tangent	Thickness (mm)
F4BM	2.2	0.0007	1
Paper	2.25	0.045	1
PET	3.4	0.07	1
